# Testicular adrenal rest tumours in an adult patient with congenital adrenal hyperplasia

**DOI:** 10.1259/bjrcr.20220072

**Published:** 2022-11-01

**Authors:** Danielle Andrea Forster, Sherief Marzouk, Indrani Chakraborti, Daniel Berney, Konrad Wolfe, Sidath H Liyanage

**Affiliations:** 1 Department of Radiology, University College London Hospital, London, United Kingdom; 2 Department of Radiology, Southend University Hospital, England, United Kingdom; 3 Endocrinology and Metabolic Medicine, Southend University Hospital, England, United Kingdom; 4 Histopathology, Royal London Hospital, Barts Health NHS Trust, England, United Kingdom; 5 Histopathology, Southend University Hospital, England, United Kingdom

## Abstract

Testicular adrenal rest tumours (TART) are found in patients with congenital adrenal hyperplasia (CAH) with the severity of testicular infiltration linearly related to the degree of enzymatic defect and subsequent compliance with treatment. We report a highly unusual case of TART in an adult patient with CAH caused by 21-hydroxylase deficiency who had not engaged with health services over a 3-year period. Typical imaging features of TART include bilateral well-defined lesions adjacent to the rete testes. However, in this rare case, the follow-up imaging found that the entirety of the testicular parenchyma had been replaced with TART and the patient had gone on to develop an adrenal nodule. As these testicular tumours are commonly misdiagnosed as primary germ tumours and tend respond well to treatment with circadian or reverse glucocorticoids, it is essential for the radiologist to be aware of both the common and more unusual imaging features appearances of TART in patients with CAH in order to facilitate early diagnosis and thus timely initiation of treatment.

## Case presentation

A 23-year-old male with a history of congenital adrenal hyperplasia (CAH) presented to the genitourinary medical clinic with right-sided testicular pain and an associated large right-sided testicular lump. He claimed to be taking prednisolone and fludrocortisone, as recommended. On clinical examination, the testes appeared diffusely swollen and were enlarged and nodular on palpation. He did not show any clinical signs of hyperandrogenism or iatrogenic Cushings syndrome. Blood profile demonstrated elevated ACTH and 17-hydroxyprogesterone levels which can be seen in untreated cases of CAH. Urea and electrolytes and 9-am testosterone were normal. These findings suggest at least partially adequate glucocorticoid treatment. The patient was sent for an ultrasound to further characterise the clinical findings.

**Figure 1.**
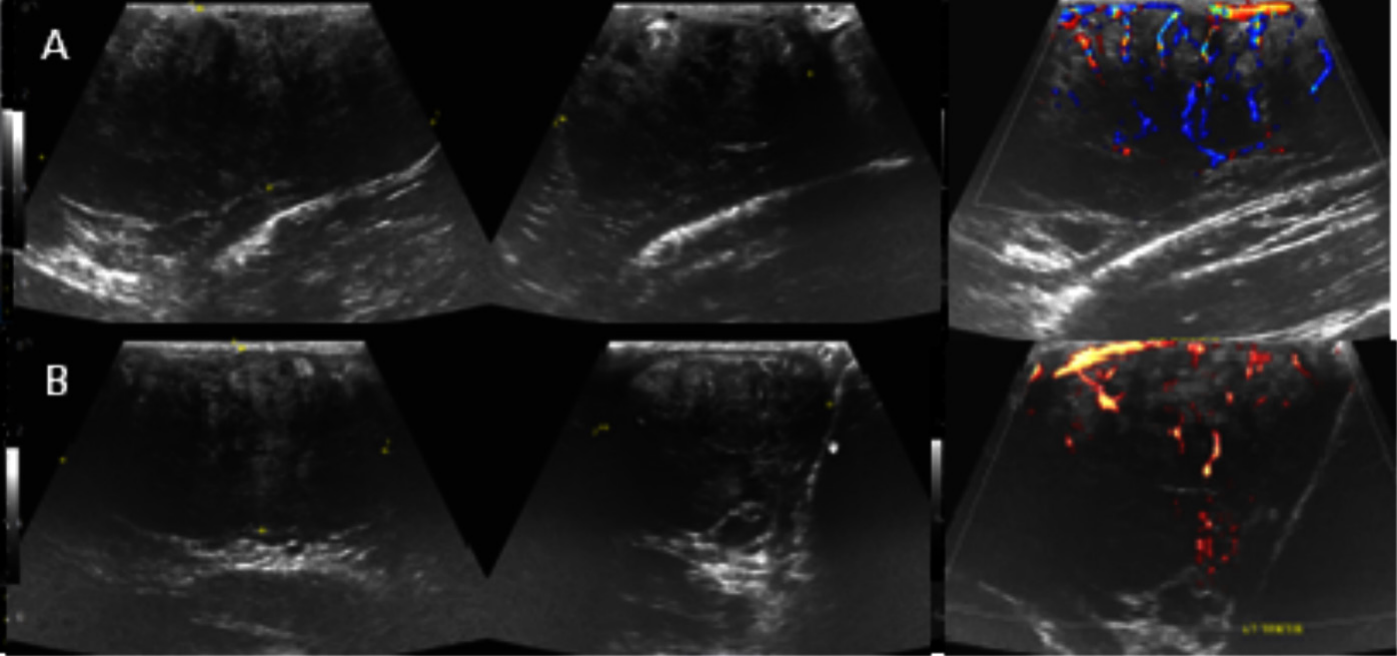
Longitudinal and transverse greyscale ultrasound and colour Doppler images of the right (**a**) and left testes (**b**). Both testes are enlarged and normal testicular parenchyma is replaced by a markedly heterogeneous and predominantly hypoechoic echotexture with disorganised increased colour Doppler flow. The margins of the abnormal parenchyma are not well circumscribed and difficult to assess on ultrasound.

A scrotal ultrasound was performed which found that the normal testicular parenchyma had been entirely replaced with ill-defined hypoechoic hypervascular masses and were grossly enlarged bilaterally (Figure 1). A differential diagnosis of lymphoma was suggested and a referral to the urology MDT was made.

**Figure 2.**
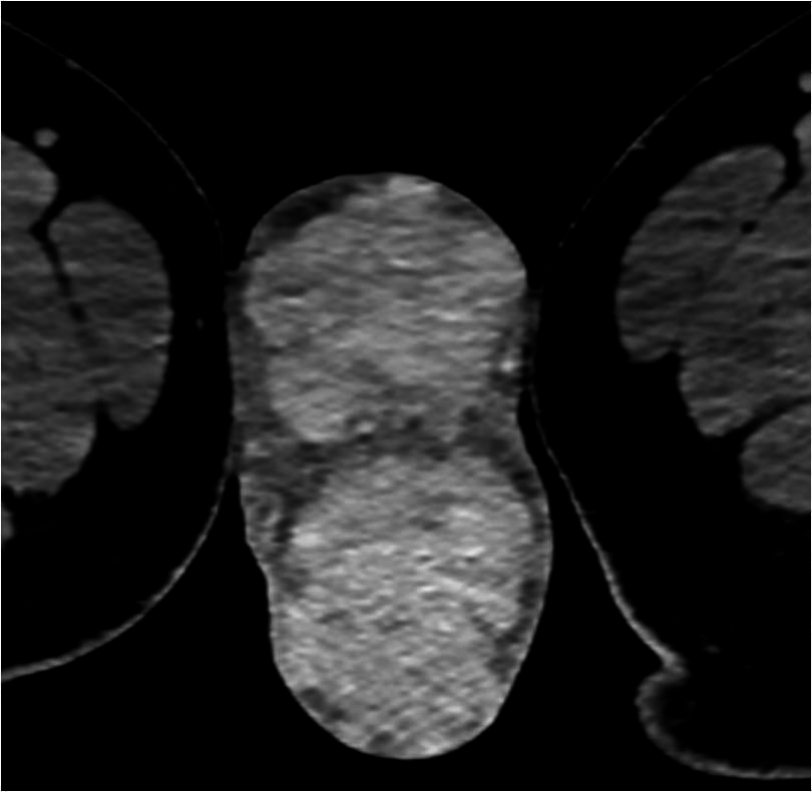
Axial contrast enhanced CT in pv phase shows avid enhancement of both testes relative to adjacent skeletal muscle. pv, portovenous

**Figure 3.**
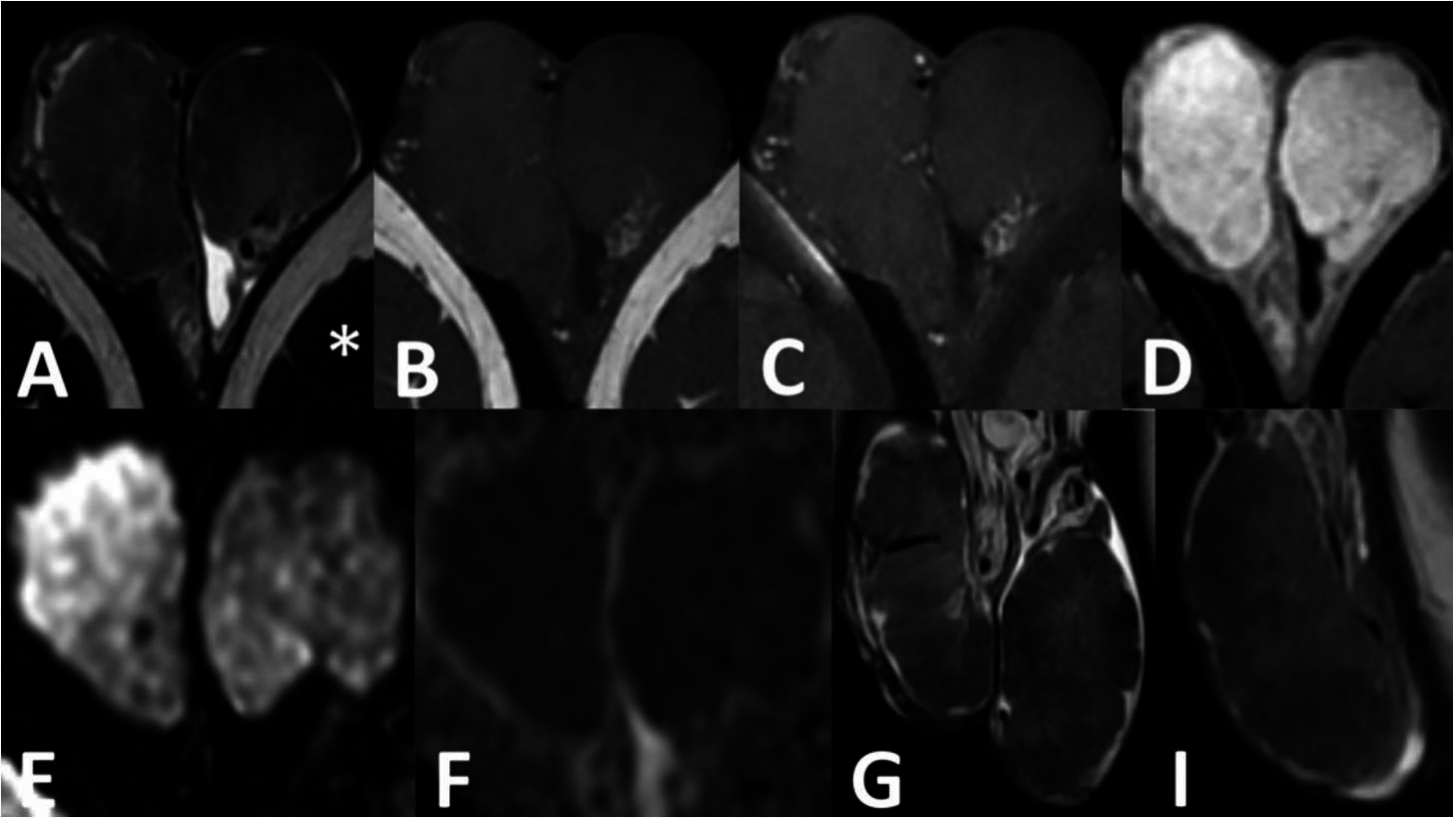
1.5 Tesla MRI (GE Signa Artist). (**A**) Axial, coronal (**G**) and sagittal *T*_2_ weighted images showing homogeneous replacement of both testes by intermediate to low T2 signal intensity—hyperintense compared to adjacent skeletal muscle (asterisk). No normal residual hyperintense T2 parenchyma or rete testis is visible. The testes are hypointense on the T1 (**b**) and T1 fat-saturation (**c**) sequences, isointense to skeletal muscle and show homogeneous intense enhancement on the dynamic post-contrast images (**d**). Both testes exhibit generalised diffusion restriction, with high signal on the b-2000 DWI images (**e**) and corresponding low signal on the ADC map (**F**). ADC, apparent diffusion coefficient; DWI, diffusion-weighted imaging.

A subsequent CT CAP was completed in order to rule out distant malignancy and although no discrete evidence of distant metastasis was found, the partially imaged testicles were hyper attenuating and avidly enhanced when compared to skeletal muscle (Figure 2). With further characterisation on MRI (Figure 3), the testicles were found to be enlarged with lobulated margins and measured 7 cm in length. There was homogenous iso- to hypointense replacement of the normal testicular parenchyma which was hyperintense when compared to skeletal muscle on both *T*_1_ and *T*_2_ weighted imaging. Furthermore, the testicles demonstrated homogenous intense enhancement on dynamic post-contrast imaging and generalised restriction of diffusion bilaterally. The paratesticular structures and penis appeared normal and there was no evidence of significant lymphadenopathy within the imaged pelvis. This appearance was thought to be most in keeping with an unusual presentation TART, however, a primary testicular germ cell tumour could not be definitively excluded on imaging and a biopsy was performed for confirmation.

**Figure 4.**
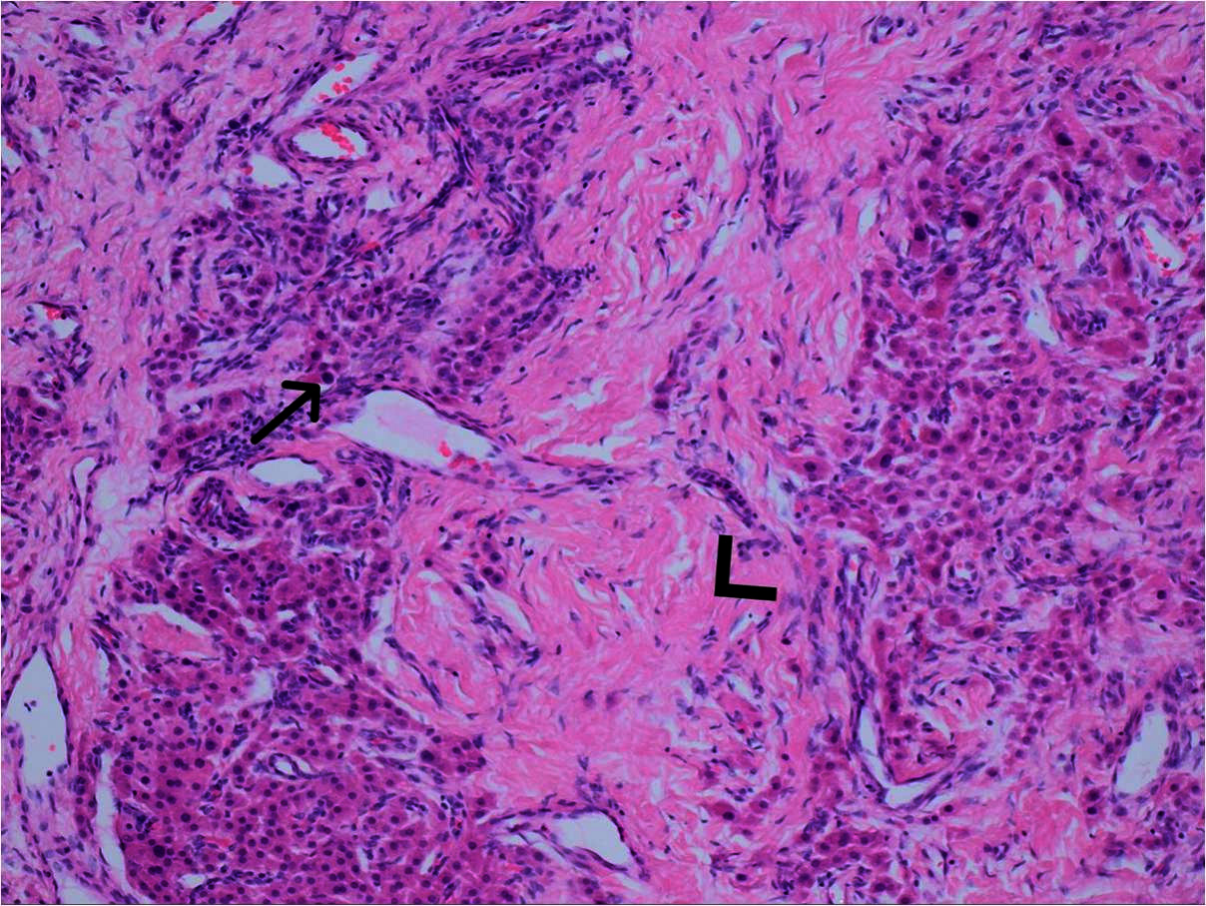
Haematoxylin and eosin stain of testicular parenchyma (100x magnification). Testicular parenchyma expanded by nests and cords of polygonal cells with round nuclei, small nucleoli and abundant eosinophilic cytoplasm (Leydig cells) [black arrow] with intervening areas of fibrotic stroma [arrow head].

Following biopsy (Figure 4), the haematoxylin and eosin stain slides revealed that the testicular parenchyma was expanded by nests and cords of large polygonal cells with intervening fibrotic stroma and patchy lymphocytic infiltrate. There was no cytological atypia, necrosis or mitotic activity identified which ruled out a potential malignant cause. The immunochemistry tested positive for Melan-A, calretinin and inhibin which, along with imaging findings and clinical history, was in keeping with a diagnosis of TART.

**Figure 5.**
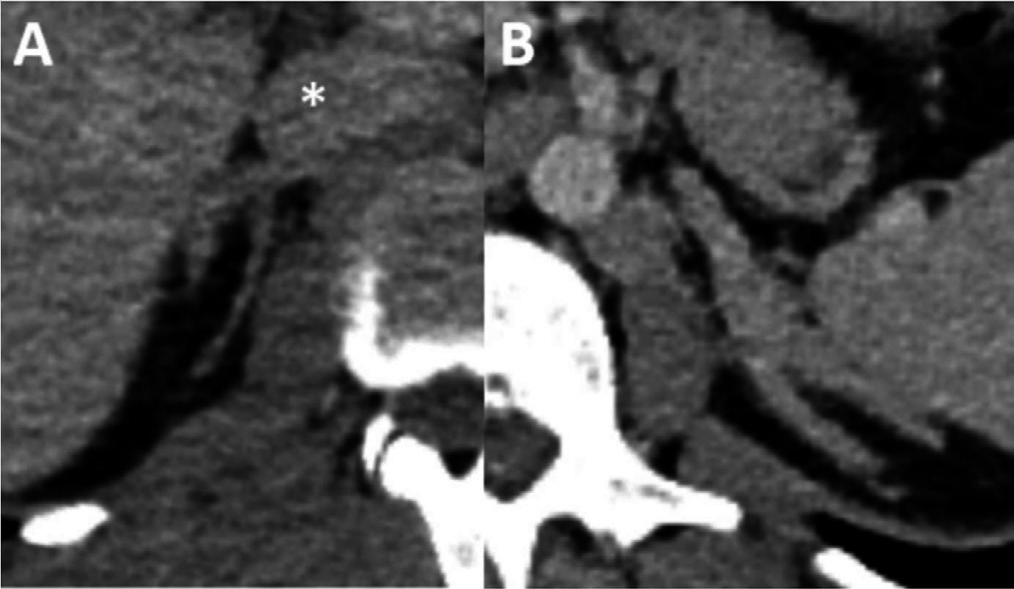
Contrast-enhanced CT (pv phase) in 2018 showing enlarged hyperplastic right (**a**) and left (**b**) adrenal glands. Asterisk in (**a**) denotes the IVC. IVC, inferior vena cava; pv, portovenous.

**Figure 6.**
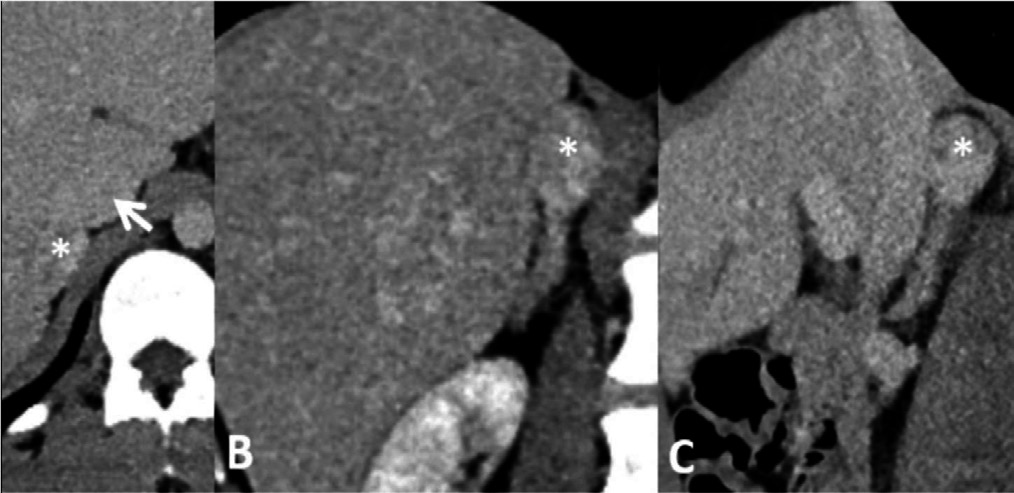
Axial (**a**), coronal (**b**) and sagittal (**c**) contrast-enhanced CT (pv phase) 3 years later showing a new 3 cm nodule (asterisk) in the body of the right adrenal gland. IVC (arrow) is noted anteromedially. IVC, inferior vena cava; pv, portovenous.

Following formal histological diagnosis and up titration of steroid treatment by the endocrinology team, the patient had a repeat testicular ultrasound 1 year later which did not demonstrate any interval change. Subsequent to this, the patient was lost to follow-up for a 3-year duration after which he presented to the accident and emergency with acute scrotal pain and oedema. At this time, the testicular imaging appearances remained relatively unchanged, however, a new 3 cm adrenal nodule arising from the body of the right adrenal gland and a hyperplastic left adrenal gland was found on CT abdomen and pelvis (Figures 5 and 6).

Unfortunately, following the CT diagnosis of new adrenal nodules, the patient failed to attend follow-up appointments and relocated.

## Discussion

CAH is a rare group of autosomal recessive disorders caused by a deficiency of the enzymes required for adrenal steroidogenesis and is seen in approximately 1 in 18,000 children born in the UK.^1^ Most frequently, CAH is caused by a deficiency of the enzyme 21-hydroxylase which is essential for cortisol and aldosterone production within the adrenal cortex.^2^.

In both male and female cases, the clinical severity of CAH is linearly correlated with the degree of enzymatic defect. Severe disease results in reduced aldosterone and cortisol production, alongside elevated androgens. Androgen excess can cause advanced bone age and short stature while aldosterone deficiency can result in salt wasting which can be fatal in some cases.^3^ In severe case of CAH, virilisation of female foetuses can be seen with male foetuses remaining anatomically normal.

Lack of cortisol synthesis—and thus low systemic levels of cortisol—results in excessive ACTH release from the anterior pituitary which in turn results in hyperstimulation of the adrenal glands. In untreated, long-term cases of excessive ACTH, the adrenal glands become hyperplastic and nodular and are at an increased risk of tumour formation such as pheochromocytomas and myelolipomas.^4^ Furthermore, the excessive systemic ACTH levels result in hyperstimulation of the adrenal rest cells which have descended with the testes during embryological development leading to the formation of testicular adrenal rest cell tumour (TART) in 95% of males with CAH.^1^ Further research has also suggested that TART may also arise from testicular pluripotent cells.^5^

Imaging plays a pivotal role in the diagnosis of TART. Ultrasound is used during the initial workup and typically reveals bilateral well-defined hypoechoic testicular masses which lie adjacent to the rete testes and usually do not demonstrate any internal vascularity on doppler ultrasound.^4^ Typical MRI features of TART include discrete lesions which are hypointense relative to normal background testicular parenchyma on *T*_2_ weighted imaging and enhance avidly with gadolinium.^6^ However, in our highly unusual case—which has not been demonstrated in any previous literature—the entirety of the normal testicular parenchyma has been replaced by TART. This is thought to be a consequence of the patients poor compliance with recommended treatment which has resulted in long-term excessive systemic ACTH levels and subsequent insidious replacement of the normal testicular parenchyma by responsive testicular adrenal rest cells. Furthermore, as seen in this case, the long-term elevated ACTH levels has resulted in bilateral adrenal hyperplasia with associated adrenal nodule formation.

In such cases of extensive infiltration of the testes, one of the main differential diagnoses that must be considered is that of lymphoma or leukaemia. However, it is more usual for lymphoma of the testes to present in older adults with the majority of cases of TART presenting in childhood and early adulthood. Sonographic features of haemotological malignancy are variable and may show discrete intratesticular hypoecheoic lesions, diffusely enlarged hypoechocic testes or parallel striated hyperechoeic and hypoechoic bands radiaging from the mediastinum on greyscale B mode imaging.^7^ Contrast-enhanced ultrasound evaluation of these lesions usually demonstrate hypervasularity.^7^ Another important differential to consider in cases of TART is that of leydig cell tumours; however, these tumours tend to present unilaterally as opposed to the bilateral nodules typically seen in TART.^8^ Leydig cell tumours can present as small, well-defined hypoechoiec intratesticular masses on B mode ultrasound. Larger lesions can show internal and peripheral colour flow whilst smaller tumours can often not demonstrate colour flow. On dynamic contrast-enhanced ultrasound, these lesions will typically demonstrate arterial phase hyperenhancement with delayed phase washout.^7^ It must be noted, however, that testicular malignancy *vs* a TART diagnosis may not be distinguishable on imaging alone. In our case with its unusual imaging appearances, it was essential that a clinical history, endocrine profile and histological diagnosis was sought in order to determine the final diagnosis.

When considering patient morbidity, the testicular parenchymal replacement seen in cases of TART can impair both spermatogenesis and testicular endocrine function with subsequent testicular atrophy and infertility in those affected. Therefore, early recognition of TART on imaging is essential in order to facilitate prompt diagnosis and initiation of treatment. TARTs have been shown to have excellent regression with steroid treatment which in selective cases can alleviate the need for surgical intervention such as orchidectomy.^6^

## Learning points

- TART are found in 94% of patients with CAH and should always be considered in the differential diagnosis when imaging the testes in such patients.
- Although TART typically appear as discrete well-defined foci adjacent to the rete testes on imaging, one must be aware of the more unusual patterns of TART especially in cases with a history of poor compliance with treatment. In this rare case, we have demonstrated that TART can insidiously replace the testicular parenchyma in its entirety
- TART can have a negative impact on patient’s quality of life and is the leading cause of infertility in males with CAH. Early recognition on imaging is therefore essential for timely initiation of treatment.
- It is not always possible to differentiate TART from malignancy on imaging alone and clinical history, endocrine profiling and histological evaluation all play essential roles in the diagnosis.
